# Associations between Mobility, Cognition, and Brain Structure in Healthy Older Adults

**DOI:** 10.3389/fnagi.2017.00155

**Published:** 2017-05-23

**Authors:** Naiara Demnitz, Enikő Zsoldos, Abda Mahmood, Clare E. Mackay, Mika Kivimäki, Archana Singh-Manoux, Helen Dawes, Heidi Johansen-Berg, Klaus P. Ebmeier, Claire E. Sexton

**Affiliations:** ^1^Department of Psychiatry, University of Oxford, Warneford HospitalOxford, United Kingdom; ^2^Oxford Centre for Functional MRI of the Brain, Nuffield Department of Clinical Neurosciences, University of OxfordJohn Radcliffe Hospital, Oxford, United Kingdom; ^3^Department of Epidemiology and Public Health, University College LondonLondon, United Kingdom; ^4^Oxford Institute of Nursing, Midwifery and Allied Health Research, Oxford Brookes UniversityOxford, United Kingdom

**Keywords:** mobility, gait, balance, aging, cognition, MRI, gray matter

## Abstract

Mobility limitations lead to a cascade of adverse events in old age, yet the neural and cognitive correlates of mobility performance in older adults remain poorly understood. In a sample of 387 adults (mean age 69.0 ± 5.1 years), we tested the relationship between mobility measures, cognitive assessments, and MRI markers of brain structure. Mobility was assessed in 2007–2009, using gait, balance and chair-stands tests. In 2012–2015, cognitive testing assessed executive function, memory and processing-speed; gray matter volumes (GMV) were examined using voxel-based morphometry, and white matter microstructure was assessed using tract-based spatial statistics of fractional anisotropy, axial diffusivity (AD), and radial diffusivity (RD). All mobility measures were positively associated with processing-speed. Faster walking speed was also correlated with higher executive function, while memory was not associated with any mobility measure. Increased GMV within the cerebellum, basal ganglia, post-central gyrus, and superior parietal lobe was associated with better mobility. In addition, better performance on the chair-stands test was correlated with decreased RD and AD. Overall, our results indicate that, even in non-clinical populations, mobility measures can be sensitive to sub-clinical variance in cognition and brain structures.

## Introduction

The capacity to move is essential for functional independence and quality of life in late adulthood. Unfortunately, mobility deteriorates with age and mobility impairments are becoming increasingly prevalent in aging populations, affecting 20–60% of adults aged 65 and older (Centers for Disease Control and Prevention, 2014; Office for National Statistics, 2015). In addition to being associated with an increased risk of falls, hospitalizations (Cesari et al., 2009), and poor quality of life (Oh et al., 2014), mounting evidence has highlighted the close relationship between aspects of mobility and cognitive processes, including executive function, memory and processing speed (Demnitz et al., 2016; Morris et al., 2016). Such findings have sparked interest in examining the neural correlates of mobility performance in older adults.

Magnetic resonance imaging (MRI) techniques allow for the examination of brain structure *in vivo* and have provided key insights into how mobility performance in aging correlates to the brain’s gray and white matter structures, both in health and disease (Holtzer et al., 2014). With regard to healthy older adults, studies examining region-specific abnormalities associated with measures of gait have implicated volume reductions within the hippocampus (Callisaya et al., 2013; Beauchet et al., 2015), the basal ganglia (Dumurgier et al., 2012), and the cerebellum (Nadkarni et al., 2014). Findings have not, however, always been consistent, as some studies have found no association between mobility measures and brain volumes in healthy older adults, or failed to replicate the previously reported association between gait measures and the cerebellum (Rosano et al., 2008; Manor et al., 2012). Although fewer studies have examined white matter structures in relation to mobility, a study using diffusion tensor imaging (DTI) to investigate white matter microstructure found that lower fractional anisotropy (FA), interpreted as decreased white matter integrity, in the genu of the corpus callosum was associated with more abnormal gait (Bhadelia et al., 2009).

Further, much of the research to date in this field has been limited to gait, a single aspect of mobility. Mobility is a multi-faceted domain that involves not only walking, but also maintaining balance and being able to rise from beds and chairs. Such additional measures (e.g., balance and chair rises) are valuable risk markers of falls (de Rekeneire et al., 2003), functional impairment (Guralnik et al., 2000), institutionalization and mortality (Cesari et al., 2009).

In a prospective cohort study, we first aim to examine the association between three objective measures of mobility (chair stands, walking time and balance) and cognitive function (memory, executive function and processing speed). Compared with gait, the relationship between balance, chair stands and cognition has been understudied, and few studies have concurrently examined multiple mobility outcomes (Demnitz et al., 2016). We aim to address these outstanding questions by analyzing these associations in a single, large, well-characterized sample of older adults. Second, we aim to explore how mobility relates to global and localized gray matter volume (GMV) and white matter integrity and test the hypothesis that poor mobility is associated with decreased GMVs and white matter integrity in a sample of older adults free from neurological illnesses.

## Materials and Methods

### Study Sample

The Whitehall II Study is a prospective cohort of British civil servants established in 1985 (Marmot and Brunner, 2005). Starting in 2012, the Whitehall II Imaging Sub-Study randomly selected participants from the Whitehall II Study for an additional assessment phase (Filippini et al., 2014). The present study sample was drawn from participants in the Whitehall II Imaging Sub-Study between May 2012 and January 2015. All included participants completed mobility assessments in 2007–2009 (Phase 9 of Whitehall II, henceforth time-point 1) and, on a subsequent phase of data collection (2012–2015, henceforth time-point 2), completed a 3T MRI brain scan and a battery of cognitive tests and health assessments. Participants were eligible if they reported no history of neurological illness, did not display significant abnormalities on structural MRI scans (e.g., evidence of infarction) and had complete data relating to mobility, cognitive, and MRI measures. Ethical approval for the Whitehall II Study was obtained from the University College London Medical School Committee on the Ethics of Human Research. The subsequent Whitehall II Imaging Sub-Study received ethical approval from the Oxford Central University Research Ethics Committee and informed written consent was obtained from all participants at all stages.

### Mobility Measures

All mobility measures were conducted at time-point 1 by a trained nurse. *Walking time* (in seconds) was measured with a stopwatch over a clearly marked 8-foot (2.44 m) course. Participants either wore closed, low-heeled, footwear or walked barefoot. Participants were instructed to walk at their own pace and to complete the course three times. In the present analysis, the quickest time was used. *Balance* was measured as time (in seconds) a balance position (one-legged stand, with eyes open) was held, with an upper cut-off of 30 s. In the *chair stands* tests, participants were asked to sit on an armless chair, rest their feet on the floor and to fold their arms across their chest. Participants were instructed to stand up and sit down without using their arms five times, and to do so as quickly as possible. The time (in seconds) taken to complete five chair rises was recorded.

### Cognitive Measures

Participants completed a battery of cognitive tests at the time of the MRI scan (time-point 2), as outlined previously (Filippini et al., 2014). The cognitive tests were classified into three domains: executive function, memory and processing speed. The executive function domain included digit span: forward, backward and sequence (Wechsler, 2008), fluency: letter and category, and the trail-making task, part B (TMT:B) (Reitan, 1955). The memory domain included the Hopkins Verbal Learning Test Revised (HVLT-R): total recall, delayed recall and recognition (Brandt, 1991), and the Rey-Osterrieth complex figure (RCF) test: immediate recall, delayed recall and recognition (Osterrieth, 1944). Finally, the processing speed domain consisted of the trail-making tasks, part A (TMT:A; Reitan, 1955), digit coding (Wechsler, 2008), and CANTAB reaction time: simple reaction time, choice reaction time, simple movement time and choice movement time (Sahakian and Owen, 1992). To ensure that higher scores always reflected better performance, signs were reversed in the trail-making and CANTAB reaction time tests.

### MRI Acquisition and Analysis

Magnetic resonance imaging data were acquired on a 3 Tesla Siemens Magnetrom Verio scanner with a 32-channel head coil at time-point 2. T1-weighted structural images were acquired using a three-dimensional rapid gradient echo sequence (2530ms repetition time, 7.37 ms echo time, 7° flip angle, 256 mm field of view and 1.0 mm isotropic voxels). Diffusion-weighted images were collected using an echoplanar imaging sequence (60 diffusion-weighted directions, *b*-value 1500 s/mm^2^; five non-diffusion weighted images, *b*-value 0s/mm^2^, with one b0 volume in the reversed phase encoded direction). Further parameters were set at: 8900 ms repetition time, 91.2 ms echo time, 192 mm field of view and 2.0 mm isotropic voxels.

Analysis of MRI data was carried out with tools from the FMRIB Software Library (FSL) (Smith et al., 2004). T1-weighted images were processed using fsl_anat^1^. Voxel-based morphometry was carried out using FSL-VBM (Douaud et al., 2007), an optimized VBM protocol (Good et al., 2001). First, brain-extracted images were gray matter-segmented using non-linear registration (Andersson et al., 2010). Resulting images were subsequently averaged to create a study-specific gray matter template. All native gray matter images were then registered to the template and modulated to correct for local expansion or contraction. The modulated gray matter images were then smoothed with an isotropic Gaussian kernel with sigma of 3 mm.

In the DTI analysis, head motion and susceptibility and eddy-current induced distortions were corrected for using the tool *eddy* (Andersson and Sotiropoulos, 2016). Voxelwise analysis of DTI data was performed using Tract-Based Spatial Statistics (TBSS) (Smith et al., 2006). First, FA images were created by fitting a diffusion tensor model to the raw diffusion data using DTIFIT. Non-brain tissue was removed using FSL’s brain extraction tool (Smith, 2002). Next, the FA data from all participants were aligned into a common space using FMRIB’s Nonlinear Registration Tool (Andersson et al., 2010). The mean FA image was then calculated and thinned to create a mean FA skeleton, which represents the centers of all tracts common to the group, yielding a mask with 128,455 voxels. Each participant’s aligned FA image was then projected onto the mean FA skeleton. Nonlinear warps and skeleton projection stages were repeated for radial diffusivity (RD) and axial diffusivity (AD) values.

### Sample Characteristics and Covariates

Age, sex, and education level were recorded for all participants at time-point 2. Education was scored on a five-point scale: (1) no qualifications, (2) GCE O-levels or equivalent, (3) GCE A-levels, college certificate or professional qualification, (4) degree, (5) higher degree.

At the time of the MRI scan, depressive symptoms, physical activity, sleep quality, BMI, blood pressure and history of arthritis were assessed as previously outlined (Filippini et al., 2014). Briefly, depressive symptoms were assessed using the Centre for Epidemiological Studies Depression Scale (CES-D), a clinically validated self-report questionnaire (Radloff, 1991). Physical activity was measured using the Community Healthy Activities Model Program for Seniors (CHAMPS) questionnaire (Stewart et al., 2001). The CHAMPS is a self-report questionnaire designed for older adults, wherein participants report the weekly frequency and duration of various activities. Each activity is assigned a Metabolic Equivalent of Task (MET) value, and the MET.Minutes used here was calculated from 20 items that represented moderate to vigorous physical activity (MET ≥ 3.0). The Pittsburgh Sleep Quality Inventory (PSQI) was used to measure sleep quality (Buysse et al., 1989) and BMI was calculated from participants’ height and weight. Finally, blood pressure was measured twice while seated (OMRON HEM-907; OMRON Healthcare UL Ltd., Milton Keynes). The average systolic and diastolic values were used to calculate mean arterial pressure (MAP; (systolic blood pressure + 2 ^∗^ diastolic blood pressure)/3). History of arthritis, defined as M00-M25 in the ICD-10, was collected through self-report.

### Statistical Analysis

To examine the association between cognition and mobility measures, statistical analysis was performed using Permutation Analysis of Linear Models (PALM) (Winkler et al., 2014). Non-parametric combination (NPC) using Fisher’s combining function was used to assess overall p-value for each cognitive domain, therefore reducing the number of comparisons (Winkler et al., 2016). Uncorrected values for individual tests were reported for descriptive purposes.

For statistical whole brain analyses of GM volumes and DTI metrics (FA, AD and RD), voxel-wise general linear models were applied using permutation-based non-parametric testing using randomize (5,000 permutations). Thresholding was carried out using TFCE (threshold-free cluster enhancement) (Smith and Nichols, 2009), and clusters were assessed for significance at *p* < 0.05, corrected for multiple comparisons across space.

In the MRI analyses of walking speed and chair stand measures, we used linear models. For balance, 278 (72%) of participants performed at ceiling – meaning they held the balance position for the whole 30 s. We therefore divided participants into two groups: good (held position for 30 s) and poor (held position for less than 30 s, average 13.8 ± 8 s) balance and conducted group comparisons in our cognitive, VBM and TBSS analyses. For our linear analyses of walking speed and chair stand measures, the VBM study template included all participants. For the group comparison analysis, a matched sub-sample of good and poor balance performers was used to create the template in accordance with FSL guidelines^2^.

All statistical analyses included age, sex, and education level as covariates. All statistical analyses were then repeated with BMI, sleep quality and history of arthritis as additional covariates.

## Results

Of the 496 Whitehall II Imaging study participants, 387 were included in our analyses (mean age 69, SD 5.11; 19% women; **Table 1**). Participants excluded due to missing data were older and less educated, but did not significantly differ to the included sample in sex distribution (Supplementary Image 1 and Table 1).

**Table 1:** Overview of sample characteristics and outcome measures.

Sample characteristics	*Mean ± SD*	Range
**Demographics**		
*N*	387	
Sex (*N*, % female)	73 (19%)	
Age (years)	69.0 ± 5.1	60.3–82.8
Education level	3.5 ± 1.1	1–5
MoCA	27.4 ± 2.2	19–30
**Mobility measures**		
Walking time (s)^∗^	2 ± 0.5	1.0–3.7
Chair stands (s)^∗^	10.3 ± 3	4.6–20.3
Balance (s)	25.4 ± 8.4	1.0–30
**Executive function**		
Digit span: Forward	11.2 ± 2.3	5–16
Digit span: Backward	9.9 ± 2.5	5–16
Digit span: Sequence	10.3 ± 2.3	0–16
Fluency: Category	22.6 ± 5.4	11–40
Fluency: Letter	15.8 ± 4.4	3–31
TMT: B (s)^∗^	64.9 ± 33.7	24–321
**Health**		
BMI	26.2 ± 4.1	17.4–42.5
Blood pressure (MAP)	97.4 ± 11.5	68.3–146.7
Depressive symptoms (CES-D)	4.6 ± 5.4	0–39
MVPA (CHAMPS; MET. Mins)	1613.9 ± 1417.6	0–7342.5
Sleep quality (PSQI)	4.6 ± 2.8	0–15
History of arthritis (*N*, %)	110 (28%)	
**Memory**		
HVLT-R: Total recall	28.1 ± 4.5	13–36
HVLT-R: Delayed recall	9.5 ± 2.5	0–12
HVLT:R: Recognition	10.8 ± 1.4	5–12
RCF: Immediate recall	15.9 ± 6.4	0–32
RCF: Delayed recall	15.5 ± 6	0–30
RCF: Recognition	8.6 ± 1.9	1–12
**Processing speed**		
TMT: A (s)^∗^	29.5 ± 10.2	13–84
Digit coding	63.4 ± 13	24–112
Simple: Reaction time (ms)^∗^	295.7 ± 70.8	211–824
Choice: Reaction time (ms)^∗^	331.1 ± 49.3	239–594
Simple: Movement time (ms)^∗^	265.3 ± 84.3	140–664.5
Choice: Reaction time (ms)^∗^	283.0 ± 76.5	120–633
**MRI measures**		
Global gray matter volume (GMV) (cm^3^)	557,244.7 ± 44,658.2	415,276–698,776
Global white matter volume (cm^3^)	563,438.1 ± 59,405.7	398,912–776,406
Global cerebrospinal fluid volume (cm^3^)	327,995.8 ± 53,933.2	172,836–501,199

*Values are mean ± SD. ^∗^Lower scores indicate better performance.*

### Mobility and Cognition

The *p*-values for the associations between each mobility measure and cognitive domain are presented in **Table 2**.

**Table 2:** Associations of mobility measures at time-point 1 with cognitive function and MRI measures at time-point 2 (*N* = 387).

	Chair stands (*s*)				Walking time (*s*)				Balance (*s* position held)			
	Model 1		Model 2		Model 1		Model 2		Model 1		Model 2	
	Pearson’s *r*	*p*	Pearson’s *r*	*p*	Pearson’s *r*	*p*	Pearson’s *r*	*p*	Cohen’s d	*p*	Cohen’s *d*	*p*
**Executive Function**		*0.089*		*0.121*		***0.042***		*0.051*		*0.198*		*0.238*
Digit Span: Forward	0.005	0.534	0.007	0.554	–0.071	0.072	–0.071	0.071	0.091	0.232	0.094	0.228
Digit Span: Backward	0.005	0.534	0.003	0.516	–0.066	0.098	–0.070	0.085	0.182	0.068	0.195	0.057
Digit Span: Sequence	–**0.095**	**0.029**	–0.073	0.072	–0.056	0.135	–0.035	0.239	0.113	0.177	0.059	0.322
Fluency: Category	–**0.101**	**0.019**	–**0.099**	**0.020**	–**0.093**	**0.030**	–**0.094**	**0.026**	–0.051	0.668	–0.053	0.664
Fluency: Letter	–0.059	0.120	–0.055	0.137	–0.016	0.387	–0.004	0.471	0.024	0.431	–0.008	0.535
TMT: B^∗^	0.017	0.623	0.017	0.623	–0.052	0.140	–0.053	0.135	0.091	0.218	0.093	0.218
**Memory**		*0.276*		0.295		*0.059*		0.073		*0.277*		0.318
HVLT-R: Total Recall	–0.033	0.242	–0.031	0.252	–**0.084**	**0.042**	–0.080	0.051	–0.010	0.530	–0.028	0.590
HVLT-R: Delayed Recall	–0.022	0.325	–0.018	0.348	–**0.094**	**0.029**	–**0.089**	**0.036**	0.126	0.149	0.111	0.180
HVLT-R: Recognition	0.053	0.852	0.062	0.889	–**0.112**	**0.013**	–**0.109**	**0.014**	0.003	0.486	–0.008	0.520
RCF: Immediate Recall	–0.059	0.118	–0.054	0.132	–0.011	0.414	–0.007	0.444	0.127	0.143	0.121	0.159
RCF: Delayed Recall	–0.069	0.085	–0.068	0.081	0.001	0.508	0.005	0.540	0.147	0.108	0.143	0.119
RCF: Recognition	0.037	0.773	0.031	0.736	0.015	0.613	0.012	0.588	–0.107	0.811	–0.103	0.795
**Processing Speed**		***< 0.001***		***<0.001***		***<0.001***		***0.002***		***0.016***		*0.057*
TMT: A^∗^	–**0.089**	**0.034**	–**0.088**	**0.035**	–0.034	0.244	–0.034	0.241	0.180	0.069	0.187	0.063
Digit Coding^∗^	–**0.109**	**0.009**	–**0.088**	**0.026**	–**0.105**	**0.012**	–**0.085**	**0.032**	**0.322**	**0.004**	**0.278**	**0.014**
Simple: Reaction Time^∗^	–**0.167**	**0.001**	–**0.146**	**0.003**	–**0.115**	**0.013**	–**0.097**	**0.028**	0.103	0.188	0.056	0.323
Choice: Reaction Time^∗^	–**0.164**	**0.001**	–**0.131**	**0.005**	–0.070	0.077	–0.040	0.205	**0.235**	**0.025**	0.163	0.090
Simple: Movement Time^∗^	–**0.143**	**0.004**	–**0.127**	**0.008**	–**0.168**	**0.001**	–**0.149**	**< 0.001**	0.103	0.193	0.049	0.342
Choice: Movement Time^∗^	–**0.122**	**0.010**	–0.104	0.019	–**0.187**	**0.001**	–0.169	< 0.001	0.115	0.171	0.060	0.311
**MRI measures**												
Global gray matter volume (% of TBV)	–**0.124**	**0.002**	–**0.112**	**0.005**	–0.069	0.064	–0.060	0.093	**0.235**	**0.025**	**0.215**	**0.043**
Global white matter volume (% of TBV)	0.051	0.867	0.043	0.830	–0.020	0.332	–0.016	0.362	0.124	0.152	0.115	0.173

*Model 1: Analyses are adjusted for age, sex, and education. Model 2: Analyses are adjusted for age, sex, education, arthritis, BMI, and sleep quality. ^∗^Scores have been reversed so that increasing scores indicate better performance. *P*-values for individual tests are not corrected for multiple comparisons. TBV = Total brain volume. Results are indicated in bold if *p* < 0.05.*

Non-parametric combination testing showed that performance on the walking test was significantly associated with processing speed (*p* < 0.001) and executive function (*p* = 0.042), after co-varying for age, sex, and education level. The chair stands and balance tests were also associated with processing speed (*p* < 0.001 and *p* = 0.016, respectively). With the exception of the relationship between processing speed and balance (*p* = 0.057), these associations remained significant (all *p* = < 0.05) after additional adjustment for BMI, sleep quality and history of arthritis (Model 2). No mobility measure was associated with memory (all *p* > 0.059).

### Mobility and Gray Matter

After adjusting for age, gender, and education, performance on the balance and chair stand tests (**Table 2**) were positively associated with global GMV (*p* = 0.007, *p* = 0.002, respectively), expressed as a % of total brain volume. Performance on the walking test yielded no significant relationship with global GMV. Additional adjustment for BMI, sleep quality and history of arthritis did not alter the significance of either association (Model 2; both *p* < 0.043).

**FIGURE 1:**
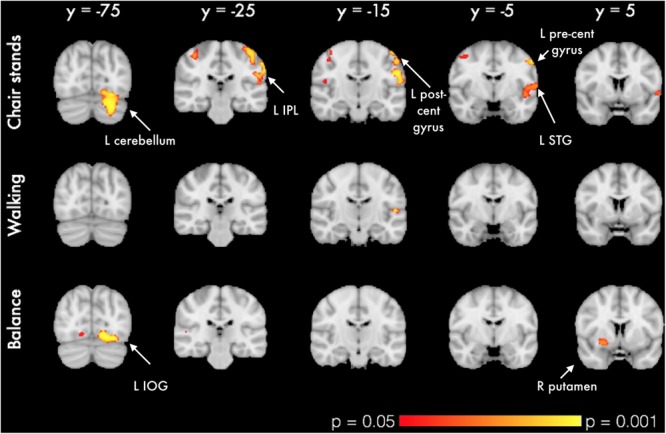
**VBM analysis of GM differences in relation to mobility measures.** Colored clusters in the first two rows represent significant positive correlations between mobility performance and GM volume. For balance (bottom row), a two-sample unpaired Student’s *t*-test was run instead (good balance vs. poor balance). Clusters represent GM areas wherein Good balance > Poor balance. All clusters (*p* < 0.05, corrected for multiple comparisons across space, with age, gender and education as covariates) are overlaid on the MNI152 template brain. Abbreviations: L IPL, Left inferior parietal lobule; L STG, Left superior temporal gyrus; L IOG, Left inferior occipital gyrus.

In voxel-wise analysis, significant mobility-related increases in voxel-wise measures of GMV were observed with better balance, chair stands and walking performances (**Figure 1**). For chair stands, clusters of significant voxels included the left post-central gyrus, the bilateral superior parietal lobule, and the left cerebellum (**Table 3**). Performance on the walking test was associated with small clusters in the left Heschl’s gyrus and the right inferior frontal gyrus. In the contrast between good balance > poor balance, clusters of significant voxels included the right putamen, the bilateral occipital fusiform gyrus and the right superior temporal gyrus (**Table 3**). Additional adjustment for BMI, sleep quality and history of arthritis attenuated clusters of significant voxels for chair stands and balance (Supplementary Image 2). No voxels remained significant in the analysis with walking time after the addition of these covariates.

**Table 3:** Significant clusters of GMV positively associated with performance on mobility measures in each VBM-style analysis. Coordinates are given in MNI space.

	No. of voxels	*P-*value	*x*	*y*	*z*	Region
Chair stands	2253	0.003	–56	–18	28	L Postcentral gyrus
	1758	<0.001	–22	–82	–32	L Cerebellum (Crus I)
	461	0.016	36	–42	64	R Superior parietal lobe
	69	0.037	50	–22	12	L Heschl’s gyrus
	10	0.049	6	–60	2	R Lingual gyrus
	2	0.05	8	–70	62	R Lateral occipital cortex
	1	0.049	–16	–52	72	L Superior parietal lobe
Walking time	91	0.028	–52	–20	10	L Heschl’s gyrus
	18	0.04	60	12	18	R Inferior frontal gyrus
Balance	762	0.001	53	25	30	L Occipital fusiform gyrus
	131	0.024	31	66	32	R Putamen
	93	0.036	35	20	35	R Occipital fusiform gyrus
	3	0.049	19	49	39	R Superior temporal gyrus

### Mobility and White Matter

Mobility measures were not associated with global white matter volume (WMV; **Table 2**).

**FIGURE 2:**
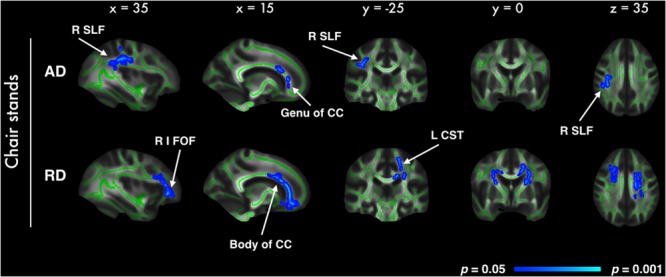
**Highlighted regions indicate significant correlations between better chair stand performance and decreased AD and RD (*p* < 0.05, after correction for multiple comparisons across space, with age, gender and education as covariates).** Significant regions are dilated for illustrative purposes and overlaid on the mean FA skeleton (green) and the mean FA image. Abbreviations: R SLF, Right superior longitudinal fasciculus; R I FOF, Right inferior fronto-occipital fasciculus; CC, Corpus callosum; L CST, Left cortico-spinal tract.

In voxel-wise analyses of DTI metrics, better performance on the chair stands test was correlated with decreased RD in 5% of all voxels, and with AD in less than 1% of all voxels. Significant voxels primarily fell within the frontal lobe, with parietal and temporal lobes also affected (**Figure 2**). For both AD and RD, tracts with significant voxels included the bilateral anterior corona radiata and the genu of the corpus callosum. No significant association was observed with FA. All analyses were adjusted for age, gender and education. No significant voxels were identified in the analysis with balance or walking time. With the addition of BMI, sleep quality and history of arthritis as covariates only the association between AD and chair stands remained significant (Supplementary Image 3).

## Discussion

In this large sample of healthy older adults, we examined how three crucial aspects of functional mobility relate to measures of cognition and MRI markers of brain structure. We found that better performance on individual mobility characteristics was associated with better processing speed (chair stands, walking time, and balance) and executive function (walking time). Contrary to previous findings (Demnitz et al., 2016), no mobility measure was found to correlate with memory. Further, mobility measures were associated with GMVs both globally (balance and chair stands) and regionally (chair stands, walking time, and balance). In addition, performance on the chair stands test was found to be correlated with indices of white matter integrity. This indicates that, even in non-clinical populations, mobility measures can be sensitive to sub-clinical variance in cognition and brain structures. The results of our analyses will be discussed in more detail next, considering each mobility measure in turn.

### Walking

The gait and cognition literature to date has highlighted the close association between the two domains, suggesting that gait may be a surrogate marker of cognitive impairment (Morris et al., 2016). Our recent meta-analysis further substantiated these findings by revealing small, yet significant, pooled effects sizes for the association between gait and executive function, processing speed and memory (Demnitz et al., 2016). Overall, our results are broadly consistent with the literature, with walking time found to be associated with executive function and processing speed in our sample. However, the relationship between faster walking and better memory only approached significance. Examination of the individual p-values for the association between each memory score and walking indicates that the walking-memory relationship may be dependent on test type. While walking relates to performance on the Hopkins Verbal Learning-Task (HVLT-R), there was no relationship with the Rey Complex Figure test. This suggests that the HVLT-R may be more sensitive for mobility studies aimed at testing cognition in older adults.

We did not find that global GM volume was associated with walking time. In voxel-wise analysis, significant voxels were limited to 2 small clusters within the right inferior frontal gyrus and the left Heschl’s gyrus. Such results for the gait-GM relationship are discordant with previously reported cross-sectional associations between smaller GM volumes and slower walking both globally (Ezzati et al., 2015) and regionally. For example, in ROI analyses, studies have found that smaller GM volume in the cerebellum (Rosano et al., 2007; Manor et al., 2012), hippocampus (Ezzati et al., 2015) and basal ganglia (Dumurgier et al., 2012) was associated with slower gait.

With regard to white matter structures, previous DTI studies have highlighted the importance of WM integrity in the corpus callosum (Bhadelia et al., 2009), the corticospinal tract (Bruijn et al., 2014) and the superior longitudinal fasciculus (Iseki et al., 2015) for healthy gait in older adults. In a large cohort of older adults with cerebral small vessel disease (SVD), de Laat et al. (2010) found significant associations between gait speed and decreased FA in frontal, temporal and occipital ROIs. Unlike previous findings, however, our study revealed no relationship between DTI metrics and walking time.

This discrepancy in findings may reflect the low level of difficulty of our walking test (2.44 m, self-paced), or its susceptibility to measurement error (manual use of stopwatch). In an analysis of longitudinal data from the InCHIANTI study, Ferrucci et al. (2016) found that, whereas a decline in the 4 m fast-speed task is already evident at 40–50 years of age, performance in the 4m usual speed walking test remains relatively stable up to the age of 65–70 years. At time-point 1, when our mobility tests were administered, our sample was, on average, 63.5 years old. It may therefore be the case that, for healthy older adults in their 60s, a 2.44 m walking test is insufficiently sensitive to detect fine differences in mobility. Further, a recent retrospective longitudinal study found that gait indices obtained from a computerized electronic walkway (GAITRite) moderated cognitive change for an additional cognitive outcome than the 3 m timed-walk test (MacDonald et al., 2017). Accordingly, more challenging walking tests (e.g., 400 m walk, or 4 m walk at fast-speed) or electronic portable walkways (e.g., GAITRite in de Laat et al., 2010) may offer more sensitive alternatives for measuring the gait-brain structure relationship.

### Balance

As previously noted, much of the mobility literature has been limited to gait. Nevertheless, age-related decline in postural control is a key predictor of falls, functional decline and quality of life in older adults. Its association with cognition, although understudied, has also been reported. Although, to the best of our knowledge, no study has reported how balance relates to each cognitive domain in the same sample, several independent studies have reported that balance is associated individually with processing speed (Rosano et al., 2005), but not memory (van Iersel et al., 2008; Verlinden et al., 2014) or executive function (Herman et al., 2011). By examining all associations in the same sample, our findings expand the existing literature and allow for a comparison across cognitive domains, providing support for the selective association with processing speed, but not memory or executive function.

In our sample, older adults with better balance had, on average, larger global GMV. In voxel-wise analysis, significant clusters included the occipital fusiform gyrus, the right putamen and the right superior temporal gyrus. Although the observed results appear to be lateralized, this should be interpreted with caution as a more bilateral pattern emerges with a slight reduction in the significance threshold (*p* < 0.1). It has been postulated that balance control declines in older adults as a result of reduced neural integration of visual, vestibular and proprioceptive feedback (Horak, 2006). The pattern of GM clusters observed in our analyses with balance appears to support this hypothesis as it indicates that good balance is associated with GMV across motor, visual, and sensorimotor regions.

Although sparse, existing findings have also linked balance performance with GM in motor and sensorimotor regions. In community-dwelling adults, it has been shown that smaller GM in the putamen, cerebellum and the right superior posterior parietal was associated with balance difficulty (Rosano et al., 2007). While our analysis did not reveal any clusters in the cerebellum or superior parietal lobe, we corroborate previous findings of smaller GM volume in the putamen in older adults with poor balance. Unlike the ROI approach adopted by Rosano et al. (2007), our use of voxel-based morphometry, a whole brain analysis technique unbiased by a priori hypotheses, may account for some of our differing results. Further, our corroborating findings with regard to the putamen are in line with the well-established role of the basal ganglia in postural control, as can be exemplified by the balance impairments observed in Parkinson’s patients with basal ganglia lesions (see Visser and Bloem, 2005 for review).

No significant voxels were identified in our TBSS analysis. In a sample of 36 healthy older adults, Van Impe et al. (2012) found that higher FA values in frontal and fronto-occipital tracts were associated with better balance performance. Although based on a small sample, these findings are particularly interesting as Van Impe et al. (2012) tested postural control using a balance platform with a movable visual surround that systematically altered levels of visual and proprioceptive feedback. Correlations between DTI metrics and postural control were only found for conditions where proprioceptive or visual feedback were compromised. This suggests that the association between balance and white matter microstructure may only be evident under more challenging conditions, and our one-legged balance test, wherein 72% of our participants performed at ceiling, did not present a challenge to most of our participants.

### Chair Stands

Performance on the chair stands test was not found to be associated with measures of executive function or memory. Processing speed, on the other hand, was significantly correlated with chair stand performance. These findings resonate with those from a large cohort study (*N* = 2,893), wherein older adults with higher scores on the Digit Symbol Substitution Test, a measure of processing speed, performed faster on the chair stand test (Rosano et al., 2005).

Few structural studies have investigated the relation between gray or white matter volumes and the ability to rise from seated. A study of community-dwelling adults (aged 35–70) found that smaller pre-frontal cortex and global GM volume were associated with slower performance on the timed-up-and go task, a measure that combines chair rising with gait (Smith et al., 2015). Although we also observed an association with global GM volume, no pre-frontal clusters emerged in our findings. However, the different nature of the mobility assessments and MRI analysis techniques used (ROI vs. VBM) limit the comparison between these results. In our VBM analysis, clusters of smaller GM associated with poorer chair stand performance included the left postcentral gyrus, the superior parietal lobe, and the cerebellum. Associations with the postcentral gyrus and the cerebellum might reflect the well-established motor function of these regions (Middleton and Strick, 2000). To navigate one’s environment, the integration of sensory and visual input is essential. Accordingly, the greater GM volume in the superior parietal lobe observed with better chair stand might reflect its role in spatial processing.

With regard to white matter, we did not find a relationship between global WM volume and chair stands. Similar results were obtained by Allali et al. (2016) in a sample of older adults referred to a memory clinic. No study has, to our knowledge, previously examined the association between DTI metrics and chair stand performance. Our voxel-wise analysis revealed increased AD and RD in frontal, parietal and temporal WM. Overall, these results suggest that indices of microstructural integrity may be important in the earlier stages of age-related decline in functional mobility measures, before volume changes are evident.

### Limitations and Conclusions

There are limitations to our study. First, included and excluded participants were significantly different in terms of age and education level. Accordingly, our sample may not be representative of the Whitehall II cohort. Second, given that the Whitehall II cohort consists of United Kingdom civil-servants from the 1980s, a working-force which was then predominantly male, our sample was not representative of both sexes (19% female). Of note, though, in our meta-analysis of the association between cognition and mobility in older adults, meta-regressions revealed no effect of sex on the cognition-mobility relationship (Demnitz et al., 2016). Nonetheless, it would be of interest for future MRI studies with a more equal sex distribution to examine the role of sex on the brain-structure-mobility relationship. Due to our large sample size it was not feasible to test all participants at the same time-of-day. Time-of-day has been shown to impact MRI measures of tissue volume (Trefler et al., 2016) and mobility performance (Bessot et al., 2015) and may therefore have been a confounder in our analyses. Further, as previously noted, our walking test may have lacked sensitivity. Although highly relevant in clinical populations, a 2.44m walking test at usual speed may be less optimal to capture brain structure-mobility relations in healthy elderly, and more precise (quantitative gait measures by electronic walkways) or more challenging (e.g., 4 m at fast pace) measures may be more appropriate in research-settings.

It should also be noted that our sample included a wide range of ages (60–83 years), and mobility performance is very variable between 60 and 80 year-olds. To explore how the reported associations may differ between age sub-groups, we conducted a *post hoc* analysis with our sample stratified by age (60–69 years and 70+ years, see Supplementary Table 2 and Images 4, 5). Interestingly, the association observed between increased global gray matter volume and better balance and chair stands performance appears to be driven by the younger sample (60–69 years). Nonetheless, these results must be interpreted with caution as the younger strata had a much larger sample size (251 vs. 136 participants).

As previously mentioned, the mobility assessments were conducted at an earlier time-point than the MRI and cognitive measurements, and this difference in time-points is a limitation of our study. The obtained associations may, therefore, have been confounded by cognitive or MRI changes occurring in the time elapsed between the two time-points. In addition, as this was an observational study, we cannot determine causality. Finally, although we obtained measures at different time-points, with mobility measures preceding MRI and cognitive testing, the nature of this study does not lend itself for conclusions of one variable predicting the presence of the other. Accordingly, our findings show that mobility measures correlate with later measures of cognition and brain structure, but do not directly test the hypothesis that poor mobility *predicts* poor cognition or increased brain atrophy at a later time-point. Randomized-controlled trials are required to determine the directionality of the relationships presented here. Given that physical activity has been shown to reliably improve mobility (Pahor et al., 2006), it would be of great interest to determine whether improving mobility (through physical activity) is an effective approach for interventions aimed at improving brain structure and function in aging populations. Promisingly, in a small sub-sample (*N* = 24) from the LIFE intervention (Fielding et al., 2011), it has been shown that, following a 24-month physical activity intervention, sedentary older adults in the physical activity group had a larger hippocampal volume than those in the control group (Rosano et al., 2016).

Given the close ties between physical activity and mobility, our findings may also tie in with the concept of “brain reserve”. It has been hypothesized that brain reserve may account for inter-individual differences in the brain’s resilience to accelerated aging, cognitive decline and late life depression (Mercado, 2008; Freret et al., 2015). Physical exercise may increase brain reserve, which may in turn have a neuroprotective effect against cognitive decline (Chirles et al., 2017). Accordingly, in a recent cross-sectional study of 2,315 older adults, lifestyle factors, including physical activity, were shown to account for 20% of the variance in cognitive test scores (Clare et al., 2017). This line of research highlights the potential impact of modifiable lifestyle factors on cognition and brain health in later life, therefore stressing the importance of developing interventions to target these factors.

## Conclusion

Our findings indicate that objective measures of poor mobility are sensitive to indices of poorer cognitive function, particularly processing speed, and markers of decreased GMV and white matter microstructure. Across mobility measures, gray matter regions involved in motor, visuospatial and cognitive control were implicated. In terms of white matter integrity, significant voxels in the corpus callosum and frontal regions were observed in the chair stands results. Altogether, these findings highlight the multiple brain regions involved in maintaining mobility in older adults, and how widespread integration between these areas is warranted. To our knowledge, this is the first study to combine reports of global and regional associations of both gray and white matter with three functional aspects of mobility measures (walking, balance and chair stands) in healthy older adults. It is our hope that the better understanding of the underpinnings of mobility in the aging brain will drive future investigations of the causality behind these relationships and guide community interventions aimed at improving mobility and cognition in older adults.

## Author Contributions

ND and CS developed the research focus. ND analyzed the data and wrote the manuscript. EZ, AM, CM, MK, AS-M, HD, HJ-B, KE, and CS contributed to theoretical and methodological refinements and provided detailed suggestions for manuscript revisions.

## Conflict of Interest Statement

The authors declare that the research was conducted in the absence of any commercial or financial relationships that could be construed as a potential conflict of interest.

## Footnotes

1 http://t2fsl.fmrib.ox.ac.uk/fsl/fslwiki/fsl_anat

2 http://fsl.fmrib.ox.ac.uk/fsl/fslwiki/FSLVBM/
