# Supplementary material for: Associations between Mobility, Cognition, and Brain Structure in Healthy Older Adults
Source: Front Aging Neurosci. 2017 May 23;9:155. doi: 10.3389/fnagi.2017.00155 (PMC5440513; doi:10.3389/fnagi.2017.00155)
Supplement: Supplementary file 1 [file Table_1.pdf]

Supplementary Table 1. Sample demographics of included (complete data) and excluded (missing data) participants.

	Complete Data	Missing Data	t	p
Demographics				
N	387	167		
Age (years)	69 ± 5.1	70.6 ± 5.6	3.4	.001
Sex (N, % Female)	73 (19%)	31 (19%)	-.08	.934
Education Level	3.6 ± 1.1	3.2 ± 1.1	-3.3	.001

Values are mean ± standard deviation and *p* values are two-tailed.
