# Supplementary material for: Associations between Mobility, Cognition, and Brain Structure in Healthy Older Adults
Source: Front Aging Neurosci. 2017 May 23;9:155. doi: 10.3389/fnagi.2017.00155 (PMC5440513; doi:10.3389/fnagi.2017.00155)
Supplement: Supplementary file 2 [file Table_2.pdf]

Supplementary Table 2. Associations of mobility measures at time-point 1 with cognitive function and MRI measures at time-point 2, stratified by age (N= 251, 60-69years; N= 136, 70+years).

	**Chair stands (*s*)**				**Walking time (*s*)**				**Balance (*s* position held)**			
	**60-69 years**		**70+ years**		**60-69 years**		**70+ years**		**60-69 years**		**70+ years**	
	Pearson's r	*p*	Pearson's r	*p*	Pearson's r	*p*	Pearson's r	*p*	Cohen's d	*p*	Cohen's d	*p*
Executive Function		*0.416*		***0.006***		***0.004***		*0.775*		*0.356*		*0.230*
**Digit Span: Forward**	0.031	0.694	0.002	0.523	-0.086	0.077	-0.029	0.632	0.041	0.393	0.179	0.181
**Digit Span: Backward**	0.073	0.876	-0.091	0.145	**-0.125**	**0.021**	0.056	0.255	0.142	0.183	**0.324**	**0.050**
**Digit Span: Sequence**	-0.004	0.478	**-0.251**	**0.001**	-0.076	0.109	-0.019	0.599	0.094	0.283	0.149	0.229
**Fluency: Category**	**-0.145**	**0.011**	-0.032	0.356	**-0.200**	**<0.001**	0.126	0.067	0.026	0.442		-0.190
**Fluency: Letter**	0.063	0.843	**-0.257**	**<0.001**	-0.048	0.219	0.063	0.232	0.004	0.491	0.058	0.386
**TMT: B***	0.127	0.980	-0.082	0.153	-0.097	0.066	0.003	0.496	0.078	0.306	0.019	0.465
Memory		*0.293*		*0.406*		***0.004***		*0.853*		*0.378*		*0.307*
**HVLT-R: Total Recall**	0.008	0.553	-0.086	0.154	**-0.168**	**0.004**	-0.052	0.723	0.060	0.347	-0.067	0.630
**HVLT-R: Delayed Recall**	-0.040	0.255	0.001	0.491	**-0.166**	**0.005**	-0.022	0.595	**0.271**	**0.046**	-0.034	0.564
**HVLT-R: Recognition**	0.035	0.707	0.081	0.828	**-0.179**	**0.003**	0.009	0.454	-0.081	0.691	0.076	0.341
**RCF: Immediate Recall**	-0.082	0.097	-0.052	0.270	-0.064	0.153	-0.092	0.875	0.038	0.405	0.212	0.134
**RCF: Delayed Recall**	-0.074	0.122	-0.076	0.185	-0.022	0.364	-0.057	0.753	0.013	0.468	0.283	0.070
**RCF: Recognition**	0.003	0.518	0.062	0.762	-0.047	0.226	-0.130	0.939	-0.175	0.869	-0.059	0.626

Processing Speed		***0.002***		***0.003***		*0.081*		***<0.001***		***0.029***		*0.262*
**TMT: A***	-0.012	0.416	**-0.195**	**0.011**	-0.094	0.072	0.055	0.752	0.230	0.080	0.052	0.393
**Digit Coding***	**-0.077**	**0.099**	**-0.161**	**0.024**	**-0.115**	**0.029**	-0.089	0.137	0.257	0.052	0.314	0.057
**Simple: Reaction Time***	**-0.122**	**0.028**	**-0.241**	**0.003**	-0.050	0.206	**-0.250**	**0.002**	0.145	0.173	0.009	0.473
**Choice: Reaction Time***	**-0.119**	**0.027**	**-0.217**	**0.007**	0.040	0.742	**-0.250**	**0.003**	0.247	0.062	0.206	0.148
**Simple: Movement Time***	**-0.206**	**<0.001**	-0.070	0.203	-0.058	0.176	**-0.358**	**<0.001**	**0.264**	**0.049**	-0.104	0.703
**Choice: Movement Time***	**-0.130**	**0.019**	-0.128	0.069	-0.076	0.113	**-0.359**	**<0.001**	0.184	0.126	0.036	0.423
MRI measures												
**Global gray matter volume (% of TBV)**	**-0.156**	**0.006**	-0.095	0.121	-0.093	0.059	-0.039	0.318	**0.349**	**0.014**	0.074	0.350
**Global white matter volume (% of TBV)**	0.116	0.974	-0.024	0.384	-0.054	0.197	0.035	0.670	0.182	0.124	0.105	0.292

Model 1: Analyses are adjusted for age, sex and education.
*Scores have been reversed so that increasing scores indicate better performance.
P-values for individual tests are not corrected for multiple comparisons. TBV = Total brain volume.
