# Supplementary figures and images for: Associations between Mobility, Cognition, and Brain Structure in Healthy Older Adults
Source: Front Aging Neurosci. 2017 May 23;9:155. doi: 10.3389/fnagi.2017.00155 (PMC5440513; doi:10.3389/fnagi.2017.00155)

Supplementary Image 1. Flow diagram of participant attrition.

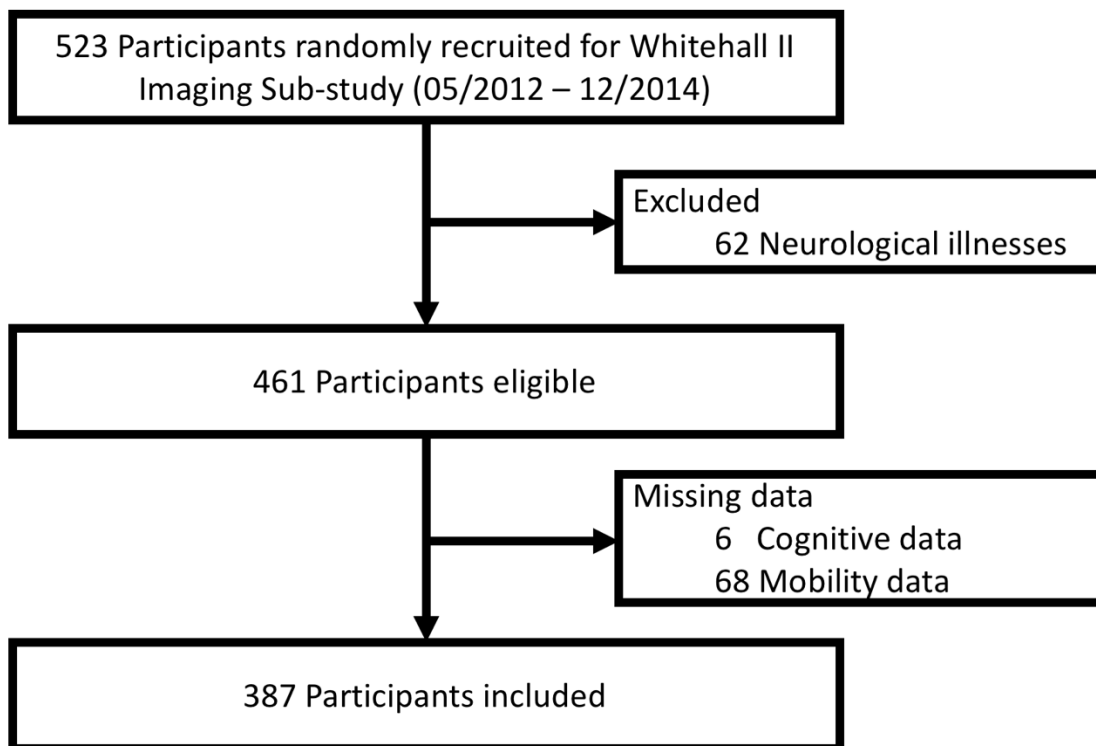

Supplement: Supplementary file 3 [file Image_1.pdf]
