# Supplementary material for: Associations between Mobility, Cognition, and Brain Structure in Healthy Older Adults
Source: Front Aging Neurosci. 2017 May 23;9:155. doi: 10.3389/fnagi.2017.00155 (PMC5440513; doi:10.3389/fnagi.2017.00155)
Supplement: Supplementary file 4 [file Image_2.pdf]

Supplementary Image 2. VBM analysis with additional covariates (Model 2).

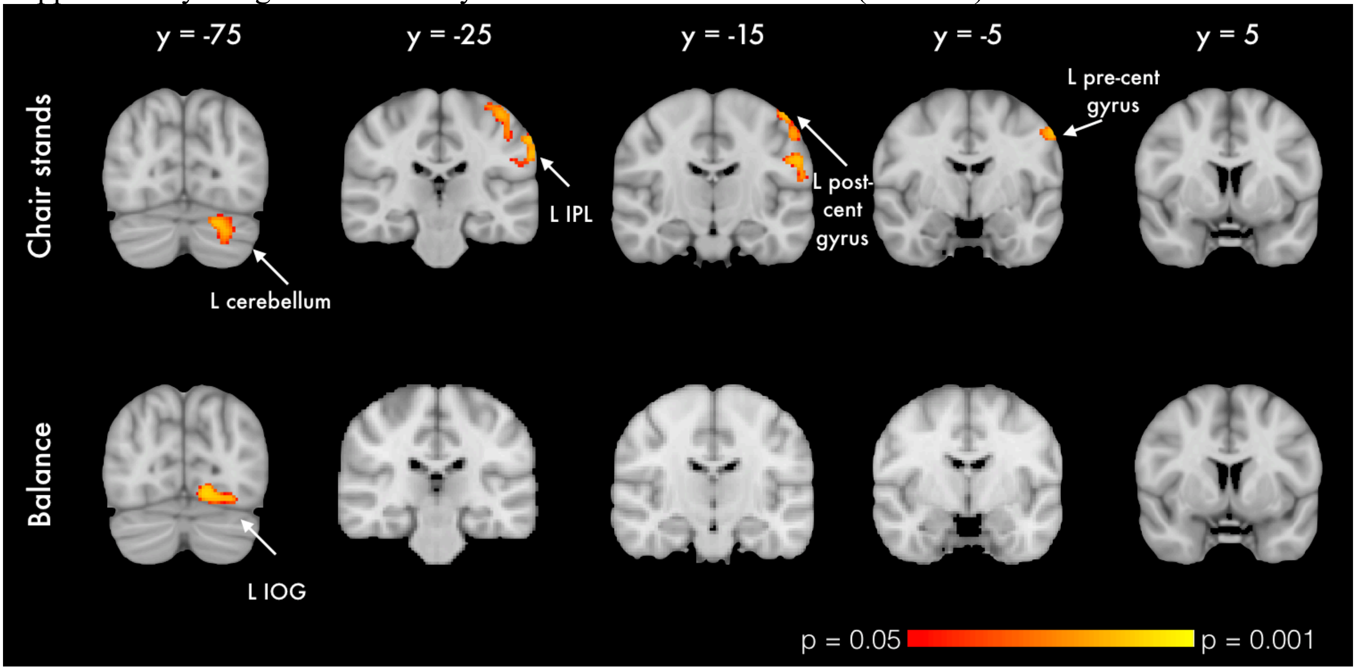

Supplementary Image 2. VBM analysis of GM differences in relation to mobility measures. Coloured clusters in the first 2 rows represent significant positive correlations between mobility performance and GM volume. For balance (bottom row), a two-sample unpaired Student's t-test was run instead (good balance vs. poor balance). Clusters represent GM areas wherein Good balance > Poor balance. All clusters ($p < 0.05$, corrected for multiple comparisons across space, controlling for age, gender, education, BMI, sleep quality and arthritis) are overlaid on the MNI152 template brain. No significant association was observed with walking time.

Abbreviations: L IPL, Left inferior parietal lobule; L IOG, Left inferior occipital gyrus.
