# Supplementary material for: Associations between Mobility, Cognition, and Brain Structure in Healthy Older Adults
Source: Front Aging Neurosci. 2017 May 23;9:155. doi: 10.3389/fnagi.2017.00155 (PMC5440513; doi:10.3389/fnagi.2017.00155)
Supplement: Supplementary file 5 [file Image_3.pdf]

Supplementary Image 3. TBSS analysis with additional covariates (Model 2).

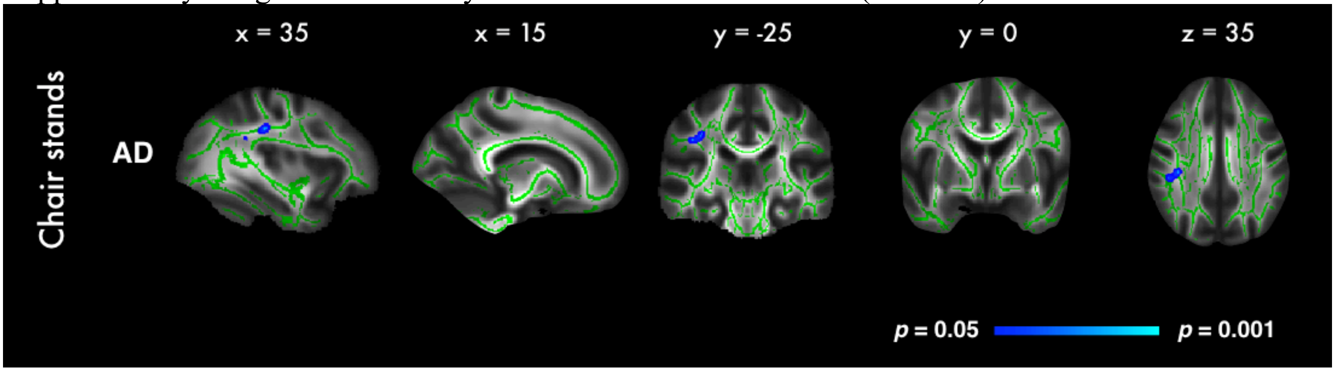

Supplementary Image 3. Highlighted regions indicate significant correlations between better chair stand performance and decreased AD ($p < 0.05$, after correction for multiple comparisons across space, with age, gender, education, BMI, sleep quality and history of arthritis as covariates). Significant regions are dilated for illustrative purposes and overlaid on the mean FA skeleton (green) and the mean FA image. No significant association was observed with FA, RD or other mobility measures.
