# Supplementary material for: Associations between Mobility, Cognition, and Brain Structure in Healthy Older Adults
Source: Front Aging Neurosci. 2017 May 23;9:155. doi: 10.3389/fnagi.2017.00155 (PMC5440513; doi:10.3389/fnagi.2017.00155)
Supplement: Supplementary file 6 [file Image_4.pdf]

Supplementary Image 4. VBM stratified analysis in 60-69 years (N = 251) and 70+ years (N=136) sub-samples.

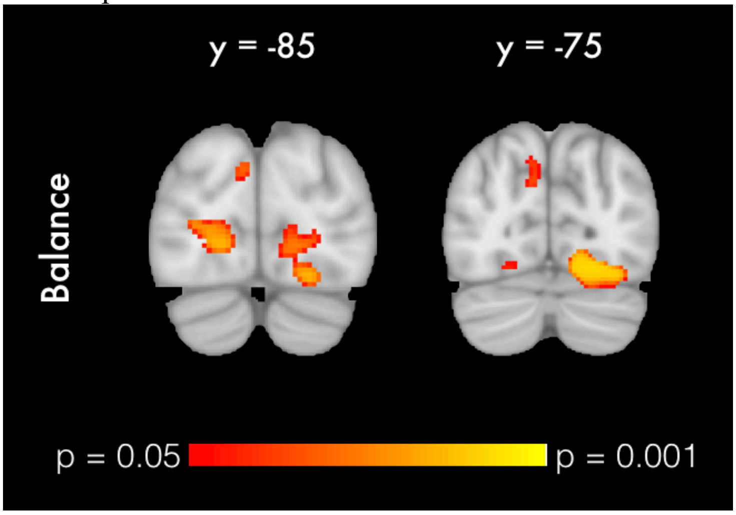

Supplementary Image 4. VBM analysis of GM differences in relation to mobility measures. Coloured clusters represent GM areas wherein Good balance > Poor balance in the 60-69 sub-sample. All clusters ($p < 0.05$, corrected for multiple comparisons across space, controlling for age, gender and education) are overlaid on the MNI152 template brain. No significant association was observed with other mobility measures or in the 70+ sub-sample.
