# Supplementary material for: Associations between Mobility, Cognition, and Brain Structure in Healthy Older Adults
Source: Front Aging Neurosci. 2017 May 23;9:155. doi: 10.3389/fnagi.2017.00155 (PMC5440513; doi:10.3389/fnagi.2017.00155)
Supplement: Supplementary file 7 [file Image_5.pdf]

Supplementary Image 5. TBSS stratified analysis in 60-69 years (N = 251) and 70+ years (N=136) sub-samples.

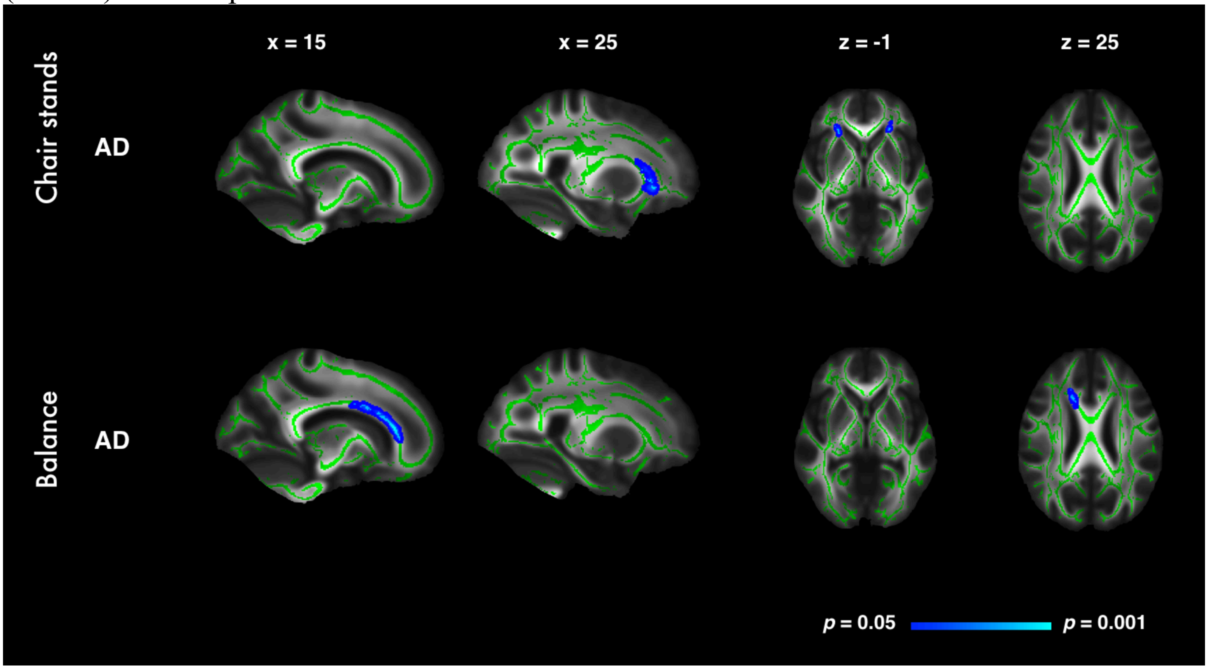


Supplementary Image 5. Highlighted regions indicate significant correlations between better chair stand and balance performance and decreased AD in the 60-69 sub-sample ($p < 0.05$, after correction for multiple comparisons across space, with age, gender and education as covariates). Significant regions are dilated for illustrative purposes and overlaid on the mean FA skeleton (green) and the mean FA image. No significant association was observed with other mobility measures or in the 70+ sub-sample.
